# Transarterial Chemoembolization of Metastatic Colorectal Carcinoma with Drug-Eluting Beads, Irinotecan (DEBIRI): Multi-Institutional Registry

**DOI:** 10.1155/2009/539795

**Published:** 2009-10-29

**Authors:** Robert C. G. Martin, Jonathan Joshi, Ken Robbins, Dana Tomalty, Ryan O'Hara, Cliff Tatum

**Affiliations:** ^1^Division of Surgical Oncology, University of Louisville School of Medicine, 315 East Broadway-Rm 311, Louisville, KY 40292, USA; ^2^Interventional Radiology Radiology, Baptist Health, 9601 Lile Dr, Radiology Consultants, Little Rock, AR 72212, USA; ^3^Interventional Radiology Radiology, Huntsville Hospital, 1963 Memorial Pkwy SW, Radiology of Huntsville, Huntsville, AL 35801, USA; ^4^Radiology & Imaging Consultants, PC, Institute for Minimally Invasive Therapy, Colorado Springs, CO 80910, USA; ^5^Norton Healthcare Radiology, 200 E Gray St, Louisville, KY 40202, USA

## Abstract

The purpose of this study was to evaluate the patient tolerance and efficacy of delivering locoregional chemotherapy to metastatic colorectal (MC) hepatic metastases via hepatic trans-arterial approach using irinotecan loaded drug eluting beads. This open-label, multi-center, single arm study included 30 MC patients, who had failed first line therapy. Of the 57 total embolization sessions, 12 (21% of sessions) were associated with adverse reactions during or after the treatment. After a median followup of 9 months, response rates by modified RECIST were 75% at 3 months and 66% at 6 months. Hepatic trans-arterial therapy using Irinotecan loaded DC Bead^TM^ was safe and effective in the treatment of MCC as demonstrated by a minimal complication rate and acceptable tumor response.

## 1. Introduction

Hepatic metastasis of colorectal cancer (MCC) is quite common occurring at some time in 23% of all of the 190 000 colorectal patients diagnosed each year [1]. While systemic chemotherapy can slow growth and even cause regression of the size of the hepatic metastases, long-term survival without local therapy is unlikely. Surgical resection of hepatic metastases continues to remain the optimal first line treatment for hepatic colorectal metastases [2, 3]. Other therapies that have been used are transarterial chemotherapy, ethanol injection, cryotherapy, radiofrequency ablation, and microwave ablation. The role of hepatic transarterial therapy of hepatic colorectal metastases continues to evolve as the technology evolves and experience with this technique matures [4]. There have been recent reports of precision transarterial therapy in metastatic colorectal cancer with acceptable results [5, 6]. The concept of hepatic arterial precision therapy is the ability to point direct doses of chemotherapy directly to the hepatic tumors with the subsequent systemic exposure.

 Patients presenting with initial unresectable metastatic colorectal metastasis either by a number of lesions or extrahepatic metastatic disease have the benefit of systemic 5 fluorouracil-based chemotherapy with a combination of oxaliplatin and/or irinotecan and offers a high rate of response (35%–50%) and a longer median survival (15–20 months) versus historical observation or 5 fluorouracil monotherapy alone [7–9]. However, patients who are refractory to 5FU-based systemic chemotherapy in combination with oxaliplatin rarely show a durable clinically relevant response rate for second or third line chemotherapy [10]. In a majority of patients the most common site of refractory progression is within the liver [6]. Thus, a minimally invasive hepatic-directed therapy that could potentially accentuate response rates as a monotherapy or in combination with systemic therapy is greatly needed.

Intra-arterial hepatic chemotherapy has the potential to accentuate both the palliation of patients and potentially improve quality of life time. Given that hepatic colorectal metastases are predominantly (95%–100%) fed by the hepatic arterial system has the advantage of delivering high-dose chemotherapy directly to the target lesion with minimal systemic side effects. This technique which has largely been used in the treatment of hepatocellular carcinoma is beginning to be expanded into other liver-dominant metastatic disease [5, 6].

 Thus, the aim of this study was to evaluate the initial safety and efficacy of irinotecan-loaded beads delivered by hepatic transarterial approach for the treatment of unresectable metastatic colorectal cancer.

## 2. Materials and Methods

An IRB-approved prospective multi-institutional open, noncontrolled repeat treatment registry was evaluated from January 2007 to October 2008 in which 30 patients presenting with liver dominant metastatic colon cancer (MCC) to the liver were treated with irinotecan drug eluting beads.

 Patients were included for therapy if they were 18 years of age, of any race or sex, who had histologic or radiologic proof of MCC to the liver, who were able to give informed consent and were eligible for treatment. Patients must have had an ECOG performance status score of less than or equal to 2 with a life expectancy of greater than or equal to 3 months, nonpregnant with an acceptable contraceptive in premenopausal women. Exclusion to therapy was contraindication to angiographic and selective visceral catheterization, significant extrahepatic disease, representing an imminent life-threatening outcome, greater than 75% of hepatic parenchymal involvement, severe liver dysfunction, pregnancy, and severe cardiac comorbidities. Only patients with liver dominant (defined as greater than 50% of the overall total body disease burden) were considered for treatment.

Standard pretherapy evaluation of patients with MCC included at least a three-phase CT of the abdomen and pelvis and chest roentgenogram at least one month prior to treatment, with the use of PET scanning depending on the institution and the availability of the technology for use. Prior systemic chemotherapy of any type and duration was allowed and was recorded.

Patients were followed for any treatment-related adverse experiences for 30 days after each treatment and monitored for survival for two years. All adverse events were recorded per standards and terminology set forth by the Cancer Therapy Evaluation Program Common Terminology Criteria for Adverse Events, Version 3.0. Followup assessments included a triphase CT scan of the liver within at least one to two months from the treatment completion with the evaluation of the enhancement pattern of the target lesion and tumor response rates measured according to RECIST [11], EASL [12], and modified RECIST [13] criteria.

### 2.1. Hepatic Angiogram

Diagnostic angiography was performed by the interventional radiologist and consisted of selective celiac and superior mesenteric arteriograms to evaluate the hepatic arterial anatomy. Once it was evaluated for the degree of hepatic tumor perfusion, the next step in evaluation was to limit any type of extrahepatic perfusion of the treatment. The most common branches that will lead to extrahepatic disposition of treatment are the right gastric and the gastroduodenal arteries which are controlled either prior to infusion with coil embolization or at a minimum distal catheter placement. In addition to this defining the amount of liver disease was integral to defining both the number of treatments and the type of catheter position and therapy that would be performed. For finite number of lesions defined as less than four lesions, treatment plan existed for a minimum of two dosing schedules of at least 50 mg of irinotecan to 100 mg of irinotecan loaded in one-two DC/LC bead vials of 100 to 300 microns, 300 to 500 microns, or 500 to 700 microns (every four to eight weeks). For diffuse disease a plan of a minimum of four-dosing schedule again of 50 to 100 mg (depending on the extent of tumor burden and the extent of hepatic parenchyma reserve) again is loaded into one-two DC/LC bead vials of the similar size as above with the plan for at least two treatments per lobe with again every three-to-four week dosing schedule, following toxicity, and extending the interval if toxicity was seen with again planned repeat CT scan three months from the first dose to evaluate tumor response.

Periprocedural medications including pain medications, antibiotic prophylaxis, intra-arterial lidocaine, corticosteroids, and proton pump inhibitors were all performed at the physician's discretion.

The mixing of loaded DC/LC beads was performed with nonionic contrast (approximately 50/50 dilution) prior to injection. A minimum of 10 cc contrast should be mixed with the loaded DC/LC bead to ensure smooth catheter delivery. After appropriate mixing and removal of the uneluted supernatant, a microcatheter is then placed based again on the extent of liver disease. For a finite number of lesions, the microcatheter is selectively placed (super selective) for the first DC/LC bead vial (a 100 to 300 micron size) with initial infusion with then a more proximal catheter placement into the right or left hepatic artery for the second DC/LC bead vial of the size choice (based on physician discretion). For diffuse disease a more lobar infusion again using a microcatheter into either the right or left artery, depending on the bulk of disease, is performed with either any of the three size DC/LC beads that the physician chooses to evaluate.

Slow injection of the irinotecan-loaded DC/LC beads is highly recommended to avoid reflux of embolic material. In addition to that, particular attention into identifying the cystic artery is recommended to ensure that the catheter tip passed this point to avoid extrahepatic infusion into the gallbladder. Additional embolic material is not usually followed after appropriate treatment but was up to the physician's discretion.

### 2.2. Drug Preparation

The saline suspension in the DC/LC bead microsphere (DEB; Biocompatibles UK, Surrey, UK) was removed and the beads were mixed with irinotecan solution at a dose of 50 mg per 2 mL at least four hours before the procedure depending on the dose that was planned to be delivered.

Data was censored at the last recorded patient contact if an endpoint was not reached. Recurrence was also evaluated using serological markers and PET scan. A recurrence was the reoccurrence of viable tumor by radiologic CT criteria of a vascular mass. In the event of subsequent hepatic therapy for recurrence of disease only the first procedure was used for the purposes of this study. Chi-square, Student's *t*-test, and Mann-Whitney's U-test for nominal, continuous, and ordinal variables were used to evaluate the association of independent variables to surgical complications. Proportional hazards analysis was performed on all variables found significant by univariate analysis. Relative risk (RR) with 95% confidence intervals was calculated as a measure of association. Differences of *P* < .05 were considered significant. Statistical analysis was performed using JMP software (JMP; SAS Institute Inc., Cary, NC).

## 3. Results

### 3.1. Patient and Tumor Characteristics

A total of 30 patients underwent 57 treatment sessions with the irinotecan drug-eluting beads. There were 11 women and 19 men in this study with a median age of 58 years (range 42–75). Past medical histories were significant for prior cardiac disease in seven patient, prior pulmonary disease in three patients, underlying diabetes in eight patients and prior alcohol abuse in five patients with six patients having a prior smoking history with a pack year median of 60 pack years (range 30–120). All of the study patients' past medical histories were negative for primary breast, carcinoid, renal, ovarian, melanoma, sarcoma, and lung cancers.

All patients had had their colon primary resected. Fifteen patients had undergone either prior anatomic hepatic lobectomy or RFA to treat their initial disease.

The extent of liver metastasis had a majority of patients with multiple lesions with a median number of lesions being five (range 1–20) with 28 patients having a bilobar lesion with the single largest target lesion being 5.0 cm (range 1–8 cm). The extent of liver involvement had 14 patients with less than 25% liver involvement, 15 patients with 26%–50% liver involvement, and one patient with greater than 50% liver involvement. The total target lesion size (sum of a maximum of five lesions) was 8.8 cm (range 1.0 to 24.0 cm). Sixteen patients had extrahepatic disease with 13 having involvement with small volume lung metastasis, one with two retroperitoneal lymph nodes, one with a bony metastasis at T1, and one with pelvic lymph node and splenic metastases.

### 3.2. Treatment

In review of the 57 treatment sessions, the median number of treatments per patient was one (range 1–5). The right lobe was the most common lobe treated in 42 patients, left lobe alone in 15 patients with the remaining being treated in a bilobar approach. The preprocedural dose planning was a median of 100 mg (range 50 to 200) involving a median of one vial with a range of one to two vials. The most common bead size was 100 to 300 microns in 37 patients, 300 to 500 micron beads in 25 patients, and 500 to 700 micron beads in 18 patients. Forty six of the 57 treatment sessions received 100% of their planned dose with the remaining 11 patients receiving a median of 80% of the dose planned (range 50%–90%) because of early stasis during treatment.

The degree of flow occlusion included partial stasis in 22 patients, near stasis in 20 patients, and complete stasis in 15 patients. Four patients did have a gel foam bland embolization following their bead therapy because of small aberrant posterior sector hepatic artery.

Periprocedural medications included antibiotics in 52 of 57 patients, most commonly being Kefsol, Levaquin, Flagyl, and Ciprofloxacin. The antiemetic medication was utilized in 57 of 57 patients with the most common medication being Zofran in 42 treatment sessions with the addition of Phenergan and/or Compazine based on the sensitivity of the patient. All patients were given periprocedural and postprocedural proton pump inhibitor or H2 blocker for gastritis prevention. Periprocedural hepatic intra-arterial Lidocaine was utilized prior to bead infusion in 35 of the 57 treatments, with systemic sedation being most commonly Morphine and Versed with a smaller distribution of patients receiving Dilaudid, Demerol, or Fentanyl. Postprocedure anti-inflammatory was utilized in 26 of 57 treatments, most commonly being Decadron or Toradol with a smaller percentage given oral Vicoprofen.

In an evaluation of periprocedural outcomes a majority of patients (60%) were treated as an outpatient setting (defined as less or equal to 23 hour admit) and on multivariate analysis identified that the risk for admission was found in patients who were receiving greater than or equal to their third treatment or who had embolic product followed in order to get to complete stasis after a successful bead infusion. These results potentially demonstrate that going to a complete stasis endpoint may lead to increase adverse events without potential benefits.

### 3.3. Patient Tolerance, Morbidity, and Mortality

Median length of hospital stay for each treatment procedure was 23 hours with a range of 23 hours to 3 days. Pre- and posttreatment WBC, hemoglobin, platelets, total bilirubin, creatinine, albumin, and INR values were obtained. However, some isolated values for particular patients were unable to be obtained. The most significant change between the pre- and posttreatment median lab values involved the WBC count, which increased 25.9%, from 7.2 to 9.1. Pre- and posttreatment median hemoglobin values were 12.1 and 12, respectively—representing a 0.8% decrease from pre- to posttreatment levels. Pre- and posttreatment median platelet values were 241 and 237.5, respectively—representing a 1.5% decrease from pre- to posttreatment levels. Pre- and posttreatment median bilirubin values were 0.6 and 0.7, respectively—representing a 16.7% increase from pre- to posttreatment levels. Pre- and posttreatment median creatinine values were 0.9 and 0.8, respectively—representing an 11.1% decrease from pre- to posttreatment levels. Pre- and posttreatment median albumin values were 4 and 3.8, respectively—representing a 5% decrease from pre- to posttreatment levels. Pre- and posttreatment median INR values did not change and were 1 and 1, respectively.

Of the 57 total embolization sessions, 12 (21% of sessions) were associated with adverse reactions during or after the treatment. However, because many patients had more than one treatment session, 9 patients suffered adverse reactions during or after their treatment. 4 of the 9 patients with adverse reactions had reactions that were rated as grade 3, with 1 subsequent death. However, the remaining 5 patients had adverse reactions to treatment that were only grades 1 and 2.

The most common adverse event 6 (50%) was postembolic syndrome type symptoms of nausea, vomiting, and, hypertension of either grade 1 or grade 2 in severity that required hospitalization and subsequent treatment. Other treatment-related events included port infection (grade 3), gastritis (grade 1), dehydration (grade 2), cholecystitis (grade 3), and hypertension (grade 2), that all resolved and were felt to be possibly or probably related to the irinotecan treatment.

One patient did have an adverse event of liver dysfunction/failure (grade 3 adverse reaction) the day after treatment. More specifically, the patient's bilirubin increased from baseline 2.9 to 3.4 on postoperative day 1. On postoperative day 2, the patient's bilirubin had decreased to 3.0 and subsequently this patient's liver dysfunction resolved. This reaction was thought to be possibly related to the embolization treatment.

One patient had an SAE of liver dysfunction, found 28 days after treatment. The patient expired from ongoing liver dysfunction 30 days after the SAE was observed. The treating physician felt that this event was possibly related to the treatment or device. The patient had been treated with 31 total cycles of FOLFOX, 9 cycles of FOLFIRI, and 12 cycles of Avastin and had a prior percutaneous RFA prior to bead therapy. The patient was given a single treatment of 2 vials of DC/LC Bead loaded with 200 mg Irinotecan. The patient had a 3-day hospital stay for nausea and then was discharged home without incident until he returned on day 28. Pt was 52 years of age, had a preoperative bilirubin of 1.9, INR of 2.0, and underwent treatment to the right lobe, with one vial of 300–500 um and one vial of 500–700 um size beads, through a lobar infusion with near stasis after bead infusion. His medications were cipro and flaygy after, with Zofran during the procedure, and an epidural for pain management. His liver involvement was 4 total lesions in seg 5–8, largest lesion 4.2 cm, with 26%–50% liver involvement, with total targets lesion size of 12.9 cm, with extrahepatic disease involving the pancreas, spleen, and lung.

### 3.4. Followup and Tumor Response

After a median followup of nine months on all 30 patients treated, response rate at three months by modified RECIST was seen in 75% of patients and at six months in 66% of patients. Similarly all 30 patients demonstrated a great than or equal 50% drop in their CEA levels at 3 months that was sustain at 6-month evaluation.

### 3.5. Discussion

The multidisciplinary management of metastatic colorectal is becoming far more complex and far more collaborative in the optimal treatment of patients with metastatic colorectal cancer. Given the ever increasing incidence of this disease with an estimated 90 000 to 100 000 new cases of metastatic colorectal cancer being diagnosed each year in the United States, this type of multidisciplinary management and collaboration is integral to the success and quality of life of the patient. These results presented demonstrate that precision hepatic arterial Irinotecan therapy is a safe and effective treatment in the management of patients with metastatic colorectal. These results demonstrate a minimal quality of life side effect and potential optimal response rates and, therefore, improved progression-free survival and overall survival in the management of this challenging disease. This initial pilot evaluation confirms the activity of this device in the management of colorectal cancer.

Hepatic-directed therapy in the management of metastatic colorectal cancer is a well-established therapy ranging from hepatic arterial infusion pumps [14] to conventional TACE [15] to implantable infusaports [16]. The rationale and clinical success of hepatic arterial therapy is well established but can be plagued by significant adverse events including both the need for surgical intervention [17], biliary sclerosis [18], significant systemic exposure [19], and catheter dislodgement and misplacement leading to inadvertent aqueous extrahepatic infusion. LC/DC bead loaded with Irinotecan has potential advantages that overcome all of the limitations of prior hepatic arterial-directed therapies. It is a more precision-directed device with minimal-to-no systemic side effects as has been reported in prior in vitro and in vivo studies [20, 21]. The data presented here demonstrated that we had no evidence of Irinotecan systemic adverse events and that all of our adverse events were related to the hepatic-directed therapy. All adverse events, on review, appear to be technically related that can be improved with adjustments in technique.

The most common adverse events were periprocedural pain, nausea, and hypertension. As demonstrated in this data only a subset of patients received preinfusion hepatic arterial Lidocaine. In a review of the data, this demonstrated to be a significant predictor of reduction or actually suppressing this adverse event when the use of 2 to 4 cc of 1% plain lidocaine is infused into the hepatic arterial system prior to bead infusion. It is well established that the DC/LC Irinotecan bead does lead to a small (5%–10%) chemotherapy burst on initial infusion into the liver and the surrounding parenchyma. The intra-arterial lidocaine has been demonstrated to be effective in reducing the symptoms of this burst, such that patients are able to tolerate the therapy similar to other established hepatic-directed therapies. The intra-arterial lidocaine has now become a standard of pretreatment technique when utilizing the Irinotecan bead for metastatic colorectal or any other type of metastatic disease in which the device is chosen to be utilized. Similar to that, one of other more common adverse events was periprocedural nausea, seen to a greater extent when anecdotally compared to conventional TACE or even prior Yttrium therapy. Effective management of this has now been achieved with now a pretreatment infusion of a higher dose of Zofran that is initiated approximately 15 to 30 minutes prior to procedure with the effective amelioration of this symptom through a more aggressive type of antiemetic treatment. The last, more common, adverse event seen was also hypertension which appeared to be refractory to the more established interventional drugs that are commonly used, those being lopressor and nitroprusside, and is found to be far more sensitive when vasotec is utilized when periprocedural hypertension is seen.

Thus in conclusion chemoembolization using Irinotecan-loaded bead was safe and effective in the treatment of MCC as demonstrated by a minimal complication rate, acceptable tumor response, and sustained reduction of CEA levels. Further larger studies are needed to confirm this data and establish where hepatic arterial precision chemotherapy should be utilized in the algorithm of the metastatic colorectal cancer patient.

## Figures and Tables

**Table 1:** Clinical demographic data in 30 colorectal metastasis patients treated with LC Bead.

Demographic	*n* = (%)
* *Caucasian	24 (80%)
* *African American	5 (19%)
* *Asian	1 (1%)

Age, median (range)	55 (40–75)

Gender	
* *Male	19 (63%)
* *Female	11 (47%)

Prior chemotherapy	
* *FOLFOX	25
* *FOLFIRI	11
* *Avastin	27
* *Xeloda	7
* *Erbitux	3
* *5FU	4
* *Vectibex	2

*Adverse Events in 57 total bead treatments*	
* *Cholecystititis	1 (2%) Grade 3
* *Dehydration	1 (2%) Grade 2
* *Gastritis	1 (2%) Grade 1
* *Hypertension	3 (5%) Grades 1–2
* *Infection	1 (2%) Grade 1
* *Liver dysfunction/failure	2 (4%) Grades 3,5
* *Nausea	5 (9%) Grades 1–2
* *Pain	1 (2%) Grade 1
* *Vomiting	3 (5%) Grades 1–2
